# Adolescent and early adulthood inflammation-associated dietary patterns in relation to premenopausal mammographic density

**DOI:** 10.1186/s13058-021-01449-0

**Published:** 2021-07-07

**Authors:** Nichole A. Garzia, Kara Cushing-Haugen, Thomas W. Kensler, Rulla M. Tamimi, Holly R. Harris

**Affiliations:** 1 Program in Epidemiology, Division of Public Health Sciences, Fred Hutchinson Cancer Research Center, 1100 Fairview Ave. North, Seattle, WA 98109-1024 USA; 2 Department of Epidemiology, School of Public Health, University of Washington, 3980 15th Ave. NE, Seattle, WA 98195-002 USA; 3 Department of Epidemiology, Harvard T.H. Chan School of Public Health, 677 Huntington Ave, Boston, MA 02115-6028 USA; 4 Department of Population Health Sciences, Weill Cornell Medicine, 1300 York Ave, New York, NY 10065-4805 USA

**Keywords:** Density, Breast, Diet, Pattern, Premenopausal

## Abstract

**Background:**

Adolescence and early adulthood has been identified as a critical time window for establishing breast cancer risk. Mammographic density is an independent risk factor for breast cancer that may be influenced by diet, but there has been limited research conducted on the impact of diet on mammographic density. Thus, we sought to examine the association between adolescent and early adulthood inflammatory dietary patterns, which have previously been associated with breast cancer risk, and premenopausal mammographic density among women in the Nurses’ Health Study II (NHSII).

**Methods:**

This study included control participants with premenopausal mammograms from an existing breast cancer case-control study nested within the NHSII who completed a Food Frequency Questionnaire in 1998 about their diet during high school (HS-FFQ) (n = 685) and/or a Food Frequency Questionnaire in 1991 (Adult-FFQ) when they were 27–44 years old (n = 1068). Digitized analog film mammograms were used to calculate the percent density, absolute dense, and non-dense areas. Generalized linear models were fit to evaluate the associations of a pro-inflammatory dietary pattern and the Alternative Healthy Eating Index (AHEI, an anti-inflammatory dietary pattern) with each breast density measure.

**Results:**

Significant associations were observed between an adolescent pro-inflammatory dietary pattern and mammographic density in some age-adjusted models; however, these associations did not remain after adjustment for BMI and other breast cancer risk factors. No associations were observed with the pro-inflammatory pattern or with the AHEI pattern in adolescence or early adulthood in fully adjusted models.

**Conclusions:**

To our knowledge, this is the first study to evaluate the dietary patterns during adolescence and early adulthood in relation to mammographic density phenotypes. Our findings do not support an association between adolescent and early adulthood diet and breast density in mid-adulthood that is independent of BMI or other breast cancer risk factors.

**Supplementary Information:**

The online version contains supplementary material available at 10.1186/s13058-021-01449-0.

## Background

Mid- and later life adult diet and breast cancer risk have been extensively studied, but few associations have emerged [1]. Prior research supports that exposures in the years prior to the first birth, including diet, may be more influential in terms of breast cancer risk than exposures occurring later in life [2, 3]. During adolescence, the mammary glands undergo rapid proliferation, while the terminal structures of the mammary glands differentiate only after the first birth. Thus, this period has been identified as a critical window for establishing future breast cancer risk [3]. In prior analyses in the Nurses’ Health Study II (NHSII), adolescent and early adulthood dietary patterns have been associated with breast cancer risk. For example, a pro-inflammatory dietary pattern, associated with markers of inflammation (e.g., C-reactive protein), was associated with a 35% increased risk of premenopausal breast cancer among women in the highest quintile of a pro-inflammatory adolescent dietary pattern [4], while adolescent dietary patterns that captured an overall healthy diet (e.g., the Alternative Healthy Eating Index) were inversely associated with breast cancer risk [2]. Systemic inflammation, particularly when low-grade and chronic, is likely to create an imbalance in pro- to anti-inflammatory markers, causing the former to be overexpressed and leading to increased cellular proliferation, as well as genomic instability and cellular damage [5]. This overexpression of certain pro-inflammatory markers (e.g., interleukin-6, C-reactive protein, and tumor necrosis factor α) may be associated with an increased risk in tumor growth and proliferation in the breasts [6, 7], with one possibility being an increase in estrogen production leading to increased breast density [8]. Women may be particularly susceptible to these potential impacts of inflammation during adolescence and early adulthood when the mammary glands are undergoing rapid change and proliferation. Mammographic density has consistently been identified as a strong, independent risk factor for breast cancer [9, 10]. More recently, it has been examined as a potential intermediary of associations between established breast cancer risk factors (e.g., childhood and adolescent somatotype, biopsy-confirmed benign breast disease, age at menarche, family history of breast cancer) and breast cancer, with results from a case-control study nested within the NHS/NHSII cohorts showing a significant mediation of mammographic density on the relationship between early life body size and breast cancer risk in premenopausal women [10]. Most prior epidemiologic studies have examined the impact of current adult diet on mammographic density by focusing on specific food items or groups (e.g., milk, alcohol) [11–13]; however, few studies have examined the association between dietary patterns and mammographic density [14, 15]. In the Minnesota Breast Cancer Family Study, a prospective cohort of family members (sisters, daughters, nieces, and grand-daughters) that began in 1990 [16], Tseng et al. reported a significant inverse association between a Mediterranean dietary pattern and mammographic density among current smokers; however, no association was observed among non-smokers [15]. In a cross-sectional study comparing two dietary patterns (Western and Mediterranean), Castello et al. reported a positive association between Western dietary pattern adherence and higher mammographic density among overweight-obese women (BMI ≥ 25), but no associations among women with a BMI < 25 [14]. No associations were observed between the Mediterranean diet and mammographic density. In both studies, the sample population consisted primarily of postmenopausal women with diet being assessed during adulthood. To our knowledge, no studies have examined the association between dietary patterns consumed during adolescence and later life mammographic density.

The aim of this study was to examine the association between adolescent and early adulthood dietary patterns and premenopausal mammographic density. Two different inflammatory-associated dietary patterns were examined: pro-inflammatory and the Alternative Healthy Eating Index (an anti-inflammatory dietary pattern). We also examined whether the associations between these dietary patterns differed by body mass index (BMI), an established risk factor for breast cancer that is also associated with mammographic density.

## Methods

### Study population

The NHSII is an ongoing prospective cohort that began in 1989 with 116,429 female registered nurses aged 25–42 years at baseline. Questionnaires are sent biennial to collect follow-up information on medical conditions, health, family history, and lifestyle factors [17]. Additionally, a semi-quantitative Food Frequency Questionnaire (FFQ) has been completed every 4 years starting in 1991, and information on diet during adolescence was collected in 1998 (details below). This study was approved by the institutional review boards of the Harvard T.H. Chan School of Public Health and Brigham and Women’s Hospital. Written authorization was obtained for mammography collection (described below), and completion and return of the questionnaires were considered implied consent.

The study population for this analysis was restricted to control participants from an existing breast cancer case-control study nested within the NHSII. Details on this study have been provided previously [18]. In brief, each breast cancer case was matched to participants who had not been diagnosed with breast cancer (controls) on age, menopausal status, race/ethnicity, and blood draw characteristics. Screening mammograms were received from approximately 80% of eligible women, who were identified from the original NHSII cohort between 1996 and 1999 to be cancer-free, between the ages of 32–54 years old, who provided blood samples and were premenopausal at blood collection time [18, 19]. Screening mammograms were targeted close to the years of the cohort blood collection (1996 to 1999). Further, we collected additional screening mammograms conducted around 1997 from eligible women (cases and controls) who were not in the original breast cancer nested case-control study. Women for whom screening mammograms were not obtained did not differ from those with available mammograms in regards to breast cancer risk factors [19].

### Dietary assessment

In 1997, participants were asked if they would be willing to complete a supplemental FFQ about diet during high school (HS-FFQ). The HS-FFQ was completed by 83% (n = 47,355) of women sent this questionnaire in 1998 when they were between 33 and 52 years old [20]. This 124-item HS-FFQ was specifically designed to include foods consumed during the period of 1960–1980 when the participants would have been in high school (13–18 years old). The range of food items covered in the HS-FFQ included everything from the types of beverages, fat used for cooking, consumption of dairy, breads/cereals/grains, main dishes (e.g., meatloaf, hamburger, pasta), fruits, vegetables, and snacks/desserts. Participants reported how frequently, on average, they had consumed each serving-size–specified food item during high school. Nine responses were possible, ranging from never to six or more times a day. The HS-FFQ has been previously validated comparing two prospectively collected 131-item self-administered Youth/Adolescent Questionnaires (YAQ) that were completed when an independent population was 13–18 years old and recalled adolescent diet using the HS-FFQ 10 years later [21]. The mean corrected correlation between the HS-FFQ and the YAQs was 0.58 (range = 0.04–0.88). These mean correlations were comparable to those obtained in the validations of the current diet assessment [22–25].

Early adulthood diet was assessed with the 1991 FFQ (Adult-FFQ) which collected dietary information on more than 130 food items, with nine responses possible ranging from never to six or more times per day. The reproducibility and validity of the FFQ questionnaire have been extensively assessed [22, 25, 26]. For both questionnaires (HS-FFQ and Adult-FFQ), nutrient intakes were calculated by multiplying the portion size of a single serving of each food by its reported frequency of intake, then multiplying the total amount consumed by the nutrient content of the food and summing the nutrient contributions of all food items using the US Department of Agriculture food composition data, while also taking dietary supplements into account.

### Dietary pattern identification

The pro-inflammatory dietary pattern was previously developed using reduced rank regression (RRR) to identify foods strongly related to inflammatory biomarkers (C-reactive protein [CRP], interleukin-6 [IL-6], and tumor necrosis factor alpha [TNF-alpha]) in a subset of primarily postmenopausal women in the Nurses’ Health Study (NHS) [27–29]. This pattern is characterized by a high intake of sugar-sweetened and diet soft drinks, refined grains, red and processed meat, margarine, corn, other vegetables (celery, mushrooms, green pepper, eggplant, summer squash, and mixed vegetables), and fish, and by low intake of green leafy vegetables, cruciferous vegetables, yellow vegetables, and coffee. A pro-inflammatory dietary pattern score is calculated based on summing the intake of the foods/food groups above, based on their positive and negative associations with inflammatory markers [see Additional file 1].

An anti-inflammatory diet was defined based on the Alternative Healthy Eating Index (AHEI), for which the details of this scoring method can be found elsewhere [30, 31]. In brief, the AHEI was developed based on foods consistently associated with chronic disease risk [30]. Scoring is based on 11 different components: fruit, vegetables, whole grains, sugar-sweetened beverages and fruit juices, nuts and legumes, red/processed meat, trans fat, long-chain (n-3) fats (EPA + DHA), polyunsaturated fat (PUFA), sodium, and alcohol [see Additional file 1]. Each component contributes 0 to 10 points, with 10 indicating the component recommendation was fully met. The AHEI score is determined by summing the score values across all components, where the higher the total score, the more aligned the dietary pattern is with healthy eating (score range is 0 to 110) [30].

### Mammographic density assessment

Mammographic density was calculated using digitized analog film mammograms. Details have been described previously [32]. Mammographic density was measured using a Lumisys 85 laser film scanner (Lumisys, Sunnyvale, CA, USA) based on craniocaudal views of both breasts. Two thresholds were set for each image to determine the edge of the breast and then delineate the dense areas of the breast within the original threshold region. The Cumulus software (University of Toronto, Toronto, ON, Canada) distinguishes the dense tissue from non-dense tissue [33]. From these measurements, absolute dense area (in cm^2^), absolute non-dense area (in cm^2^), and percent mammographic density area were calculated. Since density for the left and right breast have shown to be strongly correlated [33], the average density of both breasts was used for this analysis.

### Covariate data

Information on breast cancer risk factors was collected from baseline and biennial questionnaires. The baseline questionnaire (1989) was used to determine BMI at 18 years old, age at menarche, and high school physical activity. The biennial questionnaire directly preceding the mammogram date was used to determine the covariates at the time of mammogram: age, current BMI, age at first birth, parity, biopsy-confirmed benign breast disease, and first-degree family history of breast cancer.

### Analytic sample populations

**Fig. 1:**
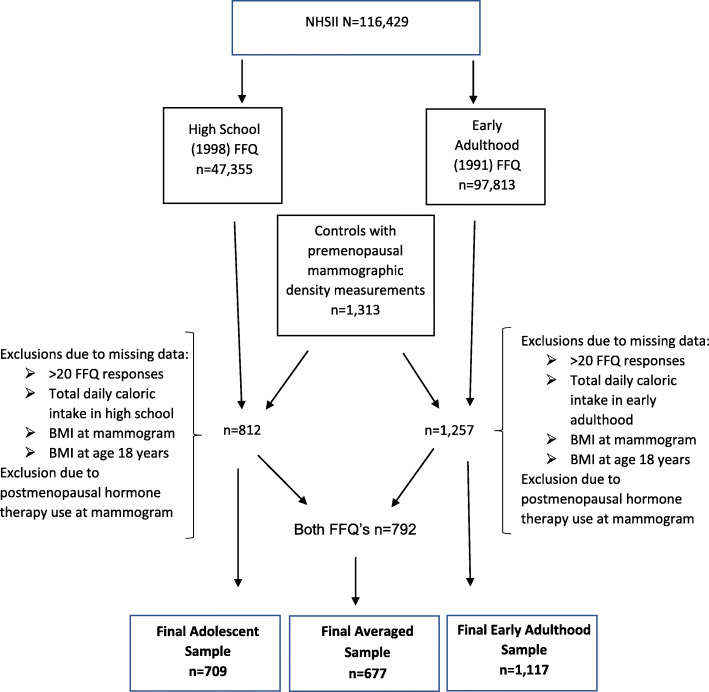
Analytic sample (adolescent, early adulthood, and averaged) identification from the Nurses’ Health Study II

Primary analyses were restricted to controls with premenopausal mammographic density measurements who completed the HS-FFQ (n = 812) and/or Adult-FFQ (n = 1257), as shown in Fig. 1. For the adolescent diet analysis, women were excluded for missing data on the HS-FFQ (> 20 blank responses, n = 3), total daily caloric intake during adolescence (n = 24), BMI at the time of mammogram (n = 29), BMI at age 18 (n = 5), and use of hormone therapy at the time of mammogram (n = 42). A total of 709 women remained for the adolescent dietary analysis following these exclusions (Fig. 1). For the early adulthood diet analysis, women were excluded for missing data on the Adult-FFQ (> 20 blank responses, n = 6), total daily caloric intake in early adulthood (n = 23), BMI at the time of mammogram (n = 45), BMI at age 18 (n = 10), and use of hormone therapy at the time of mammogram (n = 61). A total of 1117 women remained for the early adulthood dietary analysis following these exclusions (Fig. 1). For the averaged adolescent and early adulthood dietary analyses, all the above exclusions were applied resulting in a final analytic sample of 677 women for the averaged analyses. In secondary analyses, to include women who might be most sensitive to the impact of diet on mammographic density and ensure a potentially important association of interest was not missed, we included screening mammograms from breast cancer cases in addition to the controls for analytic samples of 1036 for adolescent diet and 1615 for adult diet.

### Statistical analysis

Generalized linear models were fit to evaluate the association between each quintile of each dietary pattern (pro-inflammatory and AHEI) and mammographic density phenotype (percent density, absolute dense area, non-dense area). Dietary intake was examined at three time points: adolescence, early adulthood, and average of adolescence/early adulthood. Since controls were matched to each case in the original nested case-control data [10], generalized estimating equations were used to accommodate the correlated data. The least square mean for each phenotype (percent density, absolute dense area, non-dense area) was estimated for each quintile of each dietary pattern score. Linear trends were assessed across dietary pattern quintiles for each phenotype using the Wald chi-square tests and the median value of each quintile. Sensitivity analyses were performed using square-root transformation of the mammographic density measures, but since the results were similar (data not shown), the non-transformed density measures were used as the outcomes in this study for interpretability purposes.

For each time point of dietary assessment (adolescence, early adulthood, average of adolescence/early adulthood), three models were examined: *model 1* was adjusted for age at mammogram and total caloric intake at the time of dietary assessment (high school and/or early adulthood), *model 2* was additionally adjusted for BMI at mammogram, and *model 3* was additionally adjusted for BMI at age 18, physical activity at the time of dietary assessment, alcohol intake at the time of dietary assessment, age at menarche, age at first birth, parity, history of benign breast disease, and first-degree family history of breast cancer. For the averaged analysis, physical activity and alcohol intake were adjusted using adolescent-level variables only. In addition, as BMI is strongly correlated with percent mammographic density [34–36], we conducted analyses stratified by BMI (< 25, ≥ 25). When examining the associations by BMI, tertiles of dietary patterns were used because of small numbers in cells when dietary pattern quintiles and BMI were cross-classified. Interaction effects between each dietary pattern and BMI at the time of mammogram were examined by adding an interaction term into each fully adjusted model for percent mammographic density, where BMI was treated as a dichotomous variable (< 25 and ≥ 25). Using this same approach, we also examined smoking status for potential interaction effects, since a previous study reported the associations between dietary pattern and mammographic density varied by smoking status dichotomously defined as current smokers vs. non-smokers [15].

## Results

The median age at the time of mammogram was 44 years old (range 30–55 years) with a mean percent mammographic density of 41%. The greatest overall mean caloric intakes (kcal/day) were observed among women whose dietary patterns were strongly pro-inflammatory in adolescence (Table 1) and early adulthood [see Additional file 2]. Women in the highest quintile of the pro-inflammatory dietary pattern in adolescence had the highest overall mean BMI at the time of mammogram (Table 1). The mean physical activity levels (METS/week) were highest among women who were most adherent to the AHEI in adolescence and early adulthood and among those in the lowest quintile of the pro-inflammatory dietary pattern in early adulthood.

**Table 1:** Adolescent and adult characteristics by adolescent dietary patterns among 709 premenopausal women in NHS II

Dietary pattern	Pro-inflammatory dietary pattern					Alternative Healthy Eating Index (AHEI) dietary pattern				
	Q1 (n = 144)	Q2 (n = 159)	Q3 (n = 157)	Q4 (n = 142)	Q5 (n = 107)	Q1 (n = 122)	Q2 (n = 147)	Q3 (n = 148)	Q4 (n = 156)	Q5 (n = 136)
**Adolescent characteristics**										
Caloric intake (kcal/day)	2415 (695)	2549 (698)	2774 (681)	2981 (740)	3376 (805)	2621 (733)	2638 (815)	2746 (724)	2942 (727)	2943 (864)
BMI (kg/m^2^) at age 18	20.5 (2.5)	20.5 (2.5)	21.1 (3.0)	21.4 (3.0)	22.1 (3.7)	20.4 (2.6)	21.0 (3.0)	21.4 (3.0)	21.2 (3.3)	21.4 (2.8)
Adolescent activity (METs/week)	49.6 (32.4)	47.7 (29.2)	49.7 (33.6)	44.3 (28.5)	48.5 (35.0)	47.6 (32.4)	44.0 (27.9)	43.9 (30.6)	51.1 (33.6)	53.4 (32.7)
Adolescent alcohol intake (g/day)	0.9 (3.3)	1.0 (2.6)	0.7 (2.2)	1.1 (3.5)	1.5 (7.6)	0.9 (7.0)	0.6 (2.1)	0.7 (1.8)	0.9 (2.4)	1.9 (4.8)
Age at menarche	12.5 (1.5)	12.5 (1.4)	12.3 (1.5)	12.3 (1.5)	12.3 (1.5)	12.7 (1.5)	12.5 (1.4)	12.3 (1.4)	12.2 (1.6)	12.2 (1.5)
**Adult characteristics at the time of mammogram**										
Age (years)	44.7 (4.0)	44.6 (4.0)	44.6 (3.5)	44.6 (4.2)	44.1 (4.0)	44.7 (4.4)	44.7 (3.9)	44.8 (3.8)	44.4 (3.9)	44.3 (3.6)
BMI (kg/m^2^)	24.3 (4.6)	24.8 (4.9)	25.2 (5.9)	26.3 (5.8)	27.9 (7.1)	25.2 (5.8)	25.5 (5.7)	26.1 (5.7)	25.1 (5.4)	25.8 (6.1)
Nulliparous (%)	22	17	20	14	27	16	16	21	17	27
Age at first birth*	26.8 (4.2)	27.0 (4.9)	26.4 (4.2)	26.3 (4.6)	26.2 (5.1)	26.0 (4.0)	27.0 (4.9)	26.0 (4.1)	26.6 (4.6)	27.3 (5.0)
Parity*	2.5 (1.0)	2.2 (0.9)	2.3 (1.0)	2.4 (1.0)	2.2 (0.8)	2.4 (1.1)	2.2 (0.9)	2.4 (1.0)	2.4 (0.9)	2.3 (0.8)
History of benign breast disease (%)	15	22	19	14	19	16	17	23	17	16
Family history of breast cancer (%)	6	11	10	5	7	8	7	8	7	9

*Notes*: Values are means (standard deviations) unless otherwise noted

*Among parous women only

In age-adjusted analyses, we observed as adolescent pro-inflammatory dietary pattern score increased, percent mammographic density decreased (p_trend_ = 0.005) and non-dense area increased (p_trend_ < 0.0001) (Table 2). However, these associations were no longer significant in the multivariable-adjusted models (Table 2). No associations were observed between early adulthood dietary pattern and mammographic density, or for adherence to the AHEI dietary pattern and mammographic density in adolescent or early adulthood analyses (Tables 2 and 3).

**Table 2:** Mean mammographic density phenotypes (95% confidence interval) by quintile of the adolescent dietary pattern (n = 709)

**Adolescent pro-inflammatory dietary pattern**						
	**Q1 (lowest inflammation)**	**Q2 (low inflammation)**	**Q3 (moderate inflammation)**	**Q4 (high inflammation)**	**Q5 (highest inflammation)**	**p**_**trend**_^**d**^
	**(n = 144)**	**(n = 159)**	**(n = 157)**	**(n = 142)**	**(n = 107)**	
*Percent mammographic density*						
Model 1^a^	41.7 (38.9–44.6)	42.8 (39.9–45.7)	42.8 (40.2–45.4)	40.9 (37.9–43.9)	34.8 (31.3–38.3)	0.005
Model 2^b^	39.5 (37.2–41.8)	41.4 (38.9–44.0)	42.3 (40.0–44.6)	42.3 (39.8–44.9)	39.0 (35.9–42.0)	0.93
Model 3^c^	39.6 (37.3–41.9)	41.3 (38.7–43.9)	42.0 (39.8–44.3)	42.9 (40.4–45.3)	38.7 (35.7–41.7)	0.93
*Dense area in cm*^*2*^						
Model 1^a^	43.6 (39.5–47.6)	43.5 (40.1–47.0)	43.8 (40.5–47.1)	46.8 (42.5–51.0)	41.6 (36.7–46.5)	0.97
Model 2^b^	43.4 (39.3–47.5)	43.4 (40.0–46.9)	43.7 (40.4–47.0)	46.9 (42.6–51.1)	41.9 (37.2–46.7)	0.90
Model 3^c^	43.5 (39.4–47.6)	43.0 (39.5–46.5)	43.4 (40.2–46.7)	47.7 (43.5–51.9)	41.9 (37.2–46.5)	0.78
*Non-dense area in cm*^*2*^						
Model 1^a^	66.8 (60.2–73.5)	65.1 (58.6–71.5)	66.2 (59.8–72.6)	74.8 (67.2–82.4)	92.4 (81.4–103.4)	< 0.0001
Model 2^b^	74.3 (69.6–79.0)	69.7 (64.8–74.6)	68.2 (63.2–73.2)	70.4 (65.1–75.7)	79.1 (71.4–86.8)	0.39
Model 3^c^	74.2 (69.5–78.8)	69.7 (64.8–74.7)	68.3 (63.3–73.2)	69.9 (64.6–75.1)	79.8 (72.0–87.5)	0.35
**Adolescent AHEI dietary pattern**						
	**Q1 (least healthy)**	**Q2 (unhealthy)**	**Q3 (moderately healthy)**	**Q4 (healthy)**	**Q5 (most healthy)**	**p**_**trend**_^**d**^
	**(n = 122)**	**(n = 147)**	**(n = 148)**	**(n = 156)**	**(n = 136)**	
*Percent mammographic density*						
Model 1^a^	42.2 (39.1–45.3)	41.6 (38.7–44.5)	40.2 (37.4–43.0)	40.5 (37.8–43.2)	40.7 (37.5–43.8)	0.44
Model 2^b^	41.6 (39.0–44.3)	41.6 (39.2–44.1)	41.1 (38.7–43.5)	39.8 (37.3–42.3)	41.0 (38.4–43.6)	0.51
Model 3^c^	41.0 (38.3–43.6)	41.7 (39.3–44.0)	41.0 (38.6–43.4)	40.2 (37.8–42.6)	41.4 (38.8–43.9)	0.89
*Dense area in cm*^*2*^						
Model 1^a^	42.9 (38.6–47.1)	45.7 (41.7–49.6)	43.1 (39.5–46.7)	43.4 (40.1–46.7)	44.7 (39.9–49.5)	0.83
Model 2^b^	42.8 (38.6–47.1)	45.7 (41.7–49.6)	43.2 (39.5–46.8)	43.3 (40.1–46.6)	44.7 (39.9–49.5)	0.83
Model 3^c^	42.1 (37.8–46.3)	45.8 (42.0–49.7)	43.4 (39.8–47.0)	43.5 (40.2–46.8)	44.8 (40.2–49.5)	0.66
*Non-dense area in cm*^*2*^						
Model 1^a^	65.8 (58.1–73.5)	74.5 (67.0–81.9)	70.8 (64.2–77.4)	72.5 (65.7–79.3)	74.5 (66.4–82.6)	0.25
Model 2^b^	67.7 (62.6–72.9)	74.3 (69.1–79.5)	67.7 (62.8–72.6)	75.1 (69.5–80.6)	73.8 (68.6–79.1)	0.14
Model 3^c^	68.6 (63.5–73.7)	74.3 (69.2–79.4)	68.1 (63.2–73.0)	74.4 (69.0–79.9)	73.5 (68.1–78.8)	0.27

^a^Adjusted for adolescent total calorie intake and age at the time of mammogram

^b^Additionally adjusted for BMI at the time of mammogram

^c^Additionally adjusted for BMI at age 18 (kg/m^2^; cont.), adolescent physical activity (METs/week, quartiles), adolescent alcohol intake (drinker vs. non-drinkers), age at menarche (< 12; 12; 13; 14+ years), age at first birth (AFB) and parity combined (nulliparous; AFB < 25 years, 1–2 kids; AFB 25+ years, 1–2 kids; AFB any age, 3+ kids), history of biopsy-confirmed benign breast disease (yes; no), and first-degree family history of breast cancer (yes; no)

^d^Trend test is based on the median of the category

**Table 3:** Mean mammographic density phenotypes (95% confidence interval) by quintile of early adulthood dietary patterns (n = 1117)

**Early adult pro-inflammatory dietary pattern**						
	**Q1 (lowest inflammation)**	**Q2 (low inflammation)**	**Q3 (moderate inflammation)**	**Q4 (high inflammation)**	**Q5 (highest inflammation)**	**p**_**trend**_^**d**^
	**(n = 251)**	**(n = 232)**	**(n = 217)**	**(n = 228)**	**(n = 189)**	
*Percent mammographic density*						
Model 1^a^	42.0 (39.7–44.3)	40.2 (37.7–42.6)	41.5 (39.1–43.9)	39.0 (36.8–41.2)	40.4 (38.0–42.8)	0.23
Model 2^b^	40.6 (38.6–42.7)	40.3 (38.3–42.4)	41.4 (39.4–43.5)	40.1 (38.2–42.0)	40.7 (38.6–42.8)	0.98
Model 3^c^	40.6 (38.6–42.7)	40.2 (38.2–42.2)	41.6 (39.6–43.7)	40.1 (38.2–41.9)	40.7 (38.6–42.8)	0.99
*Dense area in cm*^*2*^						
Model 1^a^	44.6 (41.6–47.6)	44.5 (40.9–48.1)	45.8 (42.6–49.0)	42.5 (39.7–45.4)	44.4 (40.5–48.4)	0.71
Model 2^b^	44.6 (41.5–47.6)	44.5 (40.9–48.1)	45.8 (42.5–49.0)	42.6 (39.8–45.4)	44.4 (40.5–48.4)	0.73
Model 3^c^	44.4 (41.3–47.5)	44.3 (40.7–47.9)	45.9 (42.7–49.2)	42.7 (39.9–45.4)	44.6 (40.7–48.5)	0.85
*Non-dense area in cm*^*2*^						
Model 1^a^	68.2 (62.7–73.7)	74.3 (68.2–80.3)	71.9 (66.0–77.7)	75.2 (69.3–81.1)	72.9 (66.3–79.4)	0.25
Model 2^b^	72.9 (68.7–77.0)	73.8 (69.5–78.0)	71.9 (67.9–75.9)	71.3 (67.2–75.4)	71.9 (67.4–76.3)	0.57
Model 3^c^	72.3 (68.2–76.4)	73.7 (69.5–77.9)	71.6 (67.5–75.6)	71.8 (67.7–75.9)	72.5 (68.1–76.8)	0.88
**Early adult AHEI dietary pattern**						
	**Q1 (least healthy)**	**Q2 (unhealthy)**	**Q3 (moderately healthy)**	**Q4 (healthy)**	**Q5 (most healthy)**	**p**_**trend**_^**d**^
	**(n = 183)**	**(n = 236)**	**(n = 218)**	**(n = 255)**	**(n = 225)**	
*Percent mammographic density*						
Model 1^a^	39.9 (37.5–42.3)	39.9 (37.7–42.0)	42.0 (39.7–44.4)	41.4 (39.2–43.6)	39.8 (37.3–42.3)	0.83
Model 2^b^	39.7 (37.8–41.6)	40.7 (38.8–42.6)	41.5 (39.5–43.5)	41.0 (39.0–43.0)	40.1 (38.1–42.2)	0.82
Model 3^c^	39.7 (37.7–41.7)	40.8 (38.9–42.7)	41.8 (39.8–43.8)	40.8 (38.8–42.7)	39.9 (37.9–42.0)	0.98
*Dense area in cm*^*2*^						
Model 1^a^	42.0 (38.8–45.1)	45.0 (41.9–48.1)	44.8 (41.5–48.1)	46.2 (43.2–49.3)	43.1 (39.8–46.3)	0.68
Model 2^b^	42.0 (38.8–45.1)	45.1 (42.0–48.2)	44.8 (41.5–48.1)	46.2 (43.1–49.3)	43.1 (39.8–46.3)	0.68
Model 3^c^	42.2 (38.8–45.6)	45.4 (42.3–48.5)	45.2 (41.9–48.6)	45.8 (42.7–48.8)	42.6 (39.2–45.9)	0.97
*Non-dense area in cm*^*2*^						
Model 1^a^	71.5 (65.1–78.0)	75.0 (69.5–80.6)	66.9 (61.8–72.0)	72.0 (66.5–77.4)	76.2 (69.6–82.8)	0.49
Model 2^b^	72.3 (68.2–76.4)	72.2 (68.4–76.0)	68.8 (65.1–72.6)	73.4 (69.6–77.3)	74.9 (70.3–79.5)	0.30
Model 3^c^	72.9 (68.7–77.1)	72.4 (68.6–76.2)	68.6 (64.8–72.4)	73.4 (69.6–77.3)	74.5 (69.8–79.1)	0.52

^a^Adjusted for early adulthood total calorie intake and age at the time of mammogram

^b^Additionally adjusted for BMI at the time of mammogram

^c^Additionally adjusted for BMI at age 18 (kg/m^2^; cont.), early adulthood physical activity (METs/week, quartiles), early adulthood alcohol intake (0 g/day; 0.1 to < 5 g/day; 5+ g/day), age at menarche (< 12; 12; 13; 14+ years), age at first birth (AFB) and parity combined (nulliparous; AFB < 25 years, 1–2 kids; AFB 25+ years, 1–2 kids; AFB any age, 3+ kids), biopsy-confirmed benign breast disease (yes; no), first-degree family history of breast cancer (yes; no)

^d^Trend test is based on the median of the category

Upon examination of individual covariates, we observed the greatest attenuation of the effect estimates occurred with adjustment for BMI at the time of mammogram. To more fully examine the impact of BMI on the dietary pattern-mammographic density association, we stratified the percent mammographic density analyses by BMI at the time of mammogram (< 25 vs. ≥ 25) (Table 4). The mean percent mammographic density estimates were lower across all tertiles of dietary pattern scores for women with a BMI ≥ 25 compared to women with a BMI < 25, as expected, but no significant association between dietary pattern and percent mammographic density was observed within either BMI strata. With the exception of the adolescent pro-inflammatory dietary pattern, statistically significant interactions between BMI and adolescent and early adulthood dietary patterns were observed (Table 4). However, this finding is likely a reflection of the strong association between BMI and mammographic density, rather than dietary pattern. Although this suggests the effect of dietary pattern on percent mammographic density could be different across BMI categories, our analyses were not powered to detect the associations between dietary patterns and percent mammographic density with strata of BMI. In our examination of smoking status (current vs. non-smokers) as having a potential interaction effect on the association between dietary pattern and percent mammographic density, we found no evidence of interaction for either dietary pattern in adolescent or early adulthood analyses. In secondary analyses, we examined the average of the adolescent and early adulthood dietary patterns in relation to mammographic density phenotypes among the women who completed both FFQs (n = 677). The results were similar to the main analyses [see Additional file 3]. Secondary analyses were also performed including the breast cancer cases in addition to the controls, and the results were not substantially different.

**Table 4:** Mean percent mammographic density by dietary pattern tertile and stratified by BMI at mammogram

Percent mammographic density	T1	T2	T3	P_**trend**_^**c**^	P_**interaction**_
**Adolescent (n = 709)**^**a**^					
**Pro-inflammatory dietary pattern**					
BMI < 25	*(n = 164)*	*(n = 151)*	*(n = 94)*		0.57
	46.9 (43.9–49.9)	52.1 (49.0–55.3)	51.8 (46.5–57.0)	0.12	
BMI ≥ 25	*(n = 85)*	*(n = 109)*	*(n = 106)*		
	29.4 (26.0–32.7)	29.0 (26.6–31.4)	31.7 (28.7–34.6)	0.32	
**AHEI dietary pattern**					
BMI < 25	*(n = 129)*	*(n = 137)*	*(n = 143)*		< 0.0001
	49.9 (46.8–53.0)	48.2 (45.6–50.8)	50.5 (48.1–53.0)	0.76	
BMI ≥ 25	*(n = 88)*	*(n = 113)*	*(n = 99)*		
	30.6 (27.7–33.6)	30.8 (28.0–33.5)	27.9 (25.0–30.9)	0.34	
**Early adulthood (n = 1117)**^**b**^					
**Pro-inflammatory dietary pattern**					
BMI < 25	*(n = 240)*	*(n = 205)*	*(n = 185)*		0.05
	48.6 (46.5–50.8)	48.8 (46.7–50.8)	46.4 (44.3–48.5)	0.18	
BMI ≥ 25	*(n = 163)*	*(n = 155)*	*(n = 169)*		
	30.8 (28.4–33.3)	31.5 (29.2–33.9)	31.2 (29.1–33.3)	0.82	
**AHEI dietary pattern**					
BMI < 25	*(n = 196)*	*(n = 201)*	*(n = 233)*		< 0.0001
	46.6 (44.6–48.6)	48.9 (47.0–50.8)	48.5 (46.2–50.7)	0.26	
BMI ≥ 25	*(n = 146)*	*(n = 167)*	*(n = 174)*		
	31.0 (28.8–33.2)	32.2 (29.9–34.5)	30.4 (28.1–32.6)	0.65	

^a^Adjusted for adolescent total calorie intake, age at the time of mammogram, BMI at the time of mammogram, BMI at age 18 (kg/m^2^; cont.), adolescent physical activity (METs/week, 0–43, > 43), adolescent alcohol intake (drinker vs. non-drinkers), age at menarche (≤ 12; 13+ years), age at first birth (AFB) and parity combined (nulliparous; AFB < 25 years, 1–2 kids; AFB 25+ years, 1–2 kids; AFB any age, 3+ kids), history of biopsy-confirmed benign breast disease (yes; no), first-degree family history of breast cancer (yes; no)

^b^Adjusted for early adulthood total calorie intake, age at the time of mammogram, BMI at the time of mammogram, BMI at age 18 (kg/m^2^; cont.), early adulthood physical activity (METs/week, 0–8, 9–26, 27+), early adulthood alcohol intake (0 g/day; 0.1 to < 5 g/day; 5+ g/day), age at menarche (< 12; 12; 13; 14+ years), age at first birth (AFB) and parity combined (nulliparous; AFB < 25 years, 1–2 kids; AFB 25+ years, 1–2 kids; AFB any age, 3+ kids), biopsy-confirmed benign breast disease (yes; no), first-degree family history of breast cancer (yes; no)

^c^Trend test is based on the median of the category

## Discussion

In this study, we did not observe significant associations between inflammation-associated dietary patterns in adolescence or early adulthood and mammographic density in models fully adjusted for breast cancer risk factors. Although significant associations were observed between an adolescent pro-inflammatory dietary pattern and mammographic density in some age-adjusted models, with a similar but non-significant pattern for the early adulthood pro-inflammatory dietary pattern, these associations did not remain after adjustment for BMI and other breast cancer risk factors. While, as expected, the distribution of percent mammographic density varied by BMI, no associations were observed between dietary patterns and mammographic density within the strata of BMI (< 25, ≥ 25).

To our knowledge, this is the first study to evaluate the relation between adolescent dietary patterns and mammographic density as prior studies have focused on individual foods/food groups and sources of macronutrients (e.g., animal fat). In previous work in the NHSII cohort, Bertrand et al. observed that adolescent animal fat intake was positively associated with premenopausal mammographic density, an association that remained after adjustment for red meat intake and adult intake of animal fat. In addition, adolescent red meat intake had a non-significant positive association with mammographic density [32]. Consistent with these results, the Dietary Intervention Study in Children (DISC) reported that higher adolescent intake of saturated fat was associated with higher breast density measured with non-contrast MRI at ages 25–29 (n = 177 women) [37]. In a study of US Chinese immigrant women (n = 201), Tseng et al. reported adolescent red meat consumption was significantly, positively associated with mammographic density in adulthood. Interestingly, the authors reported that red meat intake in this population was as much as 2.5 times lower, even for women in the highest consumption tertile, compared to consumption levels measured in Western populations, yet an association was still observed [38]. Other studies that have investigated diet in early life and breast density have reported no significant associations [39–41].

A limited number of studies have examined the dietary patterns in relation to mammographic density, with a focus on mid-life dietary patterns. In the Minnesota Breast Cancer Family Study (n = 1286), the association between a Mediterranean dietary pattern (6 items considered beneficial: vegetables, legumes, fruits and nuts, cereals, fish, and monosaturated to saturated fat ratio; 2 items considered to not be beneficial: meat and dairy; and alcohol intake) and mammographic density was examined among a sample of predominantly postmenopausal women [15]. No overall association was observed between the Mediterranean diet and mammographic density; however, this result varied by smoking status; specifically, consuming a Mediterranean diet was significantly, inversely associated with mammographic density among current smokers, while no association was observed among never smokers. The authors reported vegetables, legumes, and cereals to be the individual food items driving the inverse association among current smokers [15]. More recently, a cross-sectional study evaluated the association between two dietary patterns, Western and Mediterranean, and mammographic density. In this study, Castello et al. observed no association between adherence to a Mediterranean dietary pattern and mammographic density, while consumption of a Western (unhealthy) dietary pattern in adulthood was associated with a higher mammographic density among women with a BMI > 25 [14]. Although these studies evaluated different dietary patterns than the current study, the Western dietary pattern is akin to the pro-inflammatory dietary pattern in that both have been associated with system inflammation, while the Mediterranean dietary pattern and AHEI are anti-inflammatory and overlap in some of the included food groups.

There are some important limitations of this study to be considered. First, measurement error could have impacted the adolescent diet assessment, since participants were asked to recall diet as much as 2 to 3 decades in the past [27, 32]. However, the validity and reproducibility of the NHSII adolescent diet recall have been previously demonstrated [22, 42], and prior studies have observed statistically significant associations between adolescent dietary intake, both individual foods groups (animal fat intake) [32] and dietary patterns (inflammatory, AHEI, and prudent) [2, 4] with later life outcomes including percent mammographic density and breast cancer risk, respectively. It may be that more, not less, robust associations are detectable given the level of measurement error inherent in the retrospective adolescent diet assessment. Another limitation is that the biomarkers used to derive the pro-inflammatory pattern were not obtained during adolescence, but in postmenopausal women. Thus, the foods that were identified as associated with these biomarkers may not be as relevant during the adolescent period resulting in additional dietary exposure misclassification.

A further limitation is related to the timing of the breast density assessment. Breast density measured through a screening mammogram may not capture the most relevant breast density measurement in regard to the impact of adolescent dietary exposures. Studies have shown that breast density declines with age [43], but these changes most commonly occur around menopause, as studies have reported declines among women who are all or mostly 40 years and older [44, 45]. Krishnan et al. evaluated the correlations between mammographic measures (dense area, percent dense area, and non-dense area) over time among women (n = 970, aged 24–83 years) identified from two studies (Melbourne Collaborative Cohort Study, and the Australian Breast Cancer Family Registry population-based case-control study) and reported that within-woman correlations taken at multiple year intervals (2, 4, 6, 7, and 10 years apart) were all highly correlated with normalized estimates ≥ 0.90 after adjustment for age and BMI [46]. Given that the current study included only premenopausal women in the analysis, that density tends to decline around menopause, and that evidence supports high within-woman correlations in measurements across time, we would expect our single premenopausal mammograms to be a reliable source of mid-life density measures for evaluating their association with adolescent/early adulthood dietary patterns. Further studies are needed to better understand the factors that influence breast density measures in the adolescent and early adulthood time periods, when mammograms are not routinely taken. Breast density measured during this time period may be essential to helping us more fully understand the association between adolescent diet and breast cancer risk.

An important strength of this study was the access to digitized mammograms, and the ability to measure dietary patterns at two different time points among the study population, adolescence and early adulthood. Information on the risk factors was also available for both time points, which allowed for necessary adjustments in multivariable analyses.

## Conclusions

In conclusion, we observed that, after adjusting for covariates including BMI at mammogram, consuming a pro-inflammatory or AHEI dietary pattern during adolescence or early adulthood was not associated with premenopausal mammographic density. More research is needed to investigate early life diet in relation to breast density using prospectively collected dietary data and breast density measured prior to the age of most mammograms.

## Supplementary Information

**Additional file 1: Table S1.** Food Components of the Pro-Inflammatory and Alternative Healthy Eating Index (AHEI) Dietary Patterns

**Additional file 2: Table S2.** Adolescent and adult characteristics by early adulthood dietary patterns among 1,117 premenopausal women in NHSII

**Additional file 3: Table S3.** Mean mammographic density phenotypes (95% confidence interval) by quintile of averaged dietary patterns (n=677)

## Data Availability

Further information including the procedures to obtain and access data from the Nurses’ Health Study II is described at https://www.nurseshealthstudy.org/researchers (contact email: nhsaccess@channing.harvard.edu).

## Abbreviations

NHS/NHSII: Nurses’ Health Study/Nurses’ Health Study II
BMI: Body mass index
FFQ: Food Frequency Questionnaire
HS-FFQ: High School Food Frequency Questionnaire
Adult-FFQ: Early Adulthood Food Frequency Questionnaire
YAQ: Youth/Adolescent Questionnaire
RRR: Reduced rank regression
CRP: C-reactive protein
IL-6: Interleukin-6
TNF-alpha: Tumor necrosis factor alpha
AHEI: Alternative Healthy Eating Index
EPA + DHA: Eicosapentaenoic acid + docosahexaenoic acid
PUFA: Polyunsaturated fats
DISC: Dietary Intervention Study in Children
MRI: Magnetic resonance imaging
