# Supplementary material for: Adolescent and early adulthood inflammation-associated dietary patterns in relation to premenopausal mammographic density
Source: Breast Cancer Res. 2021 Jul 7;23:71. doi: 10.1186/s13058-021-01449-0 (PMC8261986; doi:10.1186/s13058-021-01449-0)
Supplement: Supplementary file 1 — Additional file 1: Table S1. Food Components of the Pro-Inflammatory and Alternative Healthy Eating Index (AHEI) Dietary Patterns [file 13058_2021_1449_MOESM1_ESM.docx]

**Supplementary Table 1. Food Components of the Pro-Inflammatory and Alternative Healthy Eating Index (AHEI) Dietary Patterns**

| **Pro-Inflammatory Dietary Pattern^a^** | | **AHEI Dietary Pattern^b,c^** | |
| --- | --- | --- | --- |
| **Association with Inflammatory Markers**  **(Positive [Highest inflammation] / Negative [Lowest inflammation])** | **Food Item/Groups** | **Contribution to AHEI score**  **(Stronger [Most healthy] / Weaker [Least healthy])** | **Components (foods or nutrients)** |
| Positive | Sugar-sweetened and diet soft drinks | Stronger | Fruits |
|  | Refined grains |  | Vegetables^d^ |
|  | Red and processed meat |  | Whole grains (grams/day) |
|  | Margarine |  | Nuts, legumes, and other vegetable protein^e^ |
|  | Corn |  | Long-chain (n-3) fats (EPA+DHA) |
|  | Other vegetables (celery, mushrooms, green pepper, eggplant, summer squash, and mixed vegetables) |  | Polyunsaturated Fatty Acids, PUFAs (% energy)^f^ |
|  | Fish |  |  |
| Negative | Green leafy vegetables | Weaker | Sugar-sweetened beverages or fruit juice |
|  | Cruciferous vegetables |  | Red and processed meat |
|  | Yellow vegetables |  | Trans fat |
|  | Coffee |  | Sodium (mg/day)^g^ |

^a^Pro-inflammatory dietary pattern score was calculated by summing the intake of food items/groups according to their association with inflammatory markers (positive/negative), where the greater the consumption of positively associated foods resulted in a more pro-inflammatory diet.

^b^AHEI dietary pattern score was calculated by summing assigned scores (based on serving size consumption) of each food component above, where higher consumption of stronger adherence food items corresponded with a stronger adherence to the dietary pattern overall.

^c^Alcohol consumption (drinks/day): ≥2.5 for minimum AHEI score criteria, 0.5-1.5 for maximum AHEI score criteria.

^d^Does not include potatoes.

^e^Includes tofu.

^f^Does not include EPA or DHA intake.

^g^Sodium not included in adolescent diet calculation because it was not calculated from the HS-FFQ.
