# Supplementary material for: Adolescent and early adulthood inflammation-associated dietary patterns in relation to premenopausal mammographic density
Source: Breast Cancer Res. 2021 Jul 7;23:71. doi: 10.1186/s13058-021-01449-0 (PMC8261986; doi:10.1186/s13058-021-01449-0)
Supplement: Supplementary file 2 — Additional file 2: Table S2. Adolescent and adult characteristics by early adulthood dietary patterns among 1,117 premenopausal women in NHSII [file 13058_2021_1449_MOESM2_ESM.docx]

**Supplementary Table 2. Adolescent and adult characteristics by early adulthood dietary patterns among 1,117 premenopausal women in NHSII**

| **Dietary Pattern** | **Pro-Inflammatory Dietary Pattern** | | | | | **Alternative Healthy Eating Index (AHEI) Dietary Pattern** | | | | |
| --- | --- | --- | --- | --- | --- | --- | --- | --- | --- | --- |
|  | **Q1 (n=251)** | **Q2 (n=232)** | **Q3 (n=217)** | **Q4 (n=228)** | **Q5 (n=189)** | **Q1 (n=183)** | **Q2 (n=236)** | **Q3 (n=218)** | **Q4 (n=255)** | **Q5 (n=225)** |
| **Adolescent and Early Adulthood characteristics** | | | | | | | | | | |
| Early adulthood  caloric intake (kcal/day) | 1599 (495) | 1600 (483) | 1726 (459) | 1937 (445) | 2239 (506) | 1902 (493) | 1917 (533) | 1816 (541) | 1729 (501) | 1666 (545) |
| BMI (kg/m^2^) at 18 | 21.2 (2.9) | 21.2 (2.9) | 21.0 (2.8) | 21.3 (3.1) | 20.8 (2.8) | 20.8 (2.7) | 21.2 (3.0) | 21.0 (2.8) | 21.0 (2.7) | 21.7 (3.1) |
| Early adulthood activity (METs/week) | 24.7 (27.4) | 20.4 (23.6) | 19.3 (21.7) | 16.7 (16.2) | 16.9 (18.1) | 13.8 (16.0) | 18.7 (21.3) | 17.3 (18.1) | 22.5 (23.0) | 25.2 (27.9) |
| Early adulthood alcohol intake (g/day) | 3.8 (5.5) | 4.0 (6.7) | 3.6 (6.9) | 3.1 (6.6) | 2.4 (4.3) | 2.1 (5.4) | 2.5 (5.5) | 3.1 (6.4) | 4.2 (6.6) | 5.0 (6.0) |
| Age at menarche | 12.2 (1.3) | 12.4 (1.5) | 12.4 (1.5) | 12.5 (1.5) | 12.5 (1.5) | 12.6 (1.4) | 12.4 (1.4) | 12.3 (1.5) | 12.5 (1.5) | 12.3 (1.5) |
| **Adult characteristics at time of mammogram** | | | | | | | | | | |
| Age (years) | 45.1 (3.7) | 44.3 (3.6) | 44.5 (4.1) | 44.1 (4.3) | 43.8 (4.2) | 43.8 (4.3) | 44.1 (4.2) | 44.0 (3.9) | 45.0 (3.8) | 44.8 (3.8) |
| BMI (kg/m^2^) | 25.0 (5.4) | 25.9 (5.6) | 25.8 (5.7) | 26.4 (6.4) | 25.9 (5.7) | 25.6 (5.6) | 26.3 (6.3) | 25.4 (5.6) | 25.6 (5.3) | 26.0 (6.0) |
| Nulliparous (%) | 27 | 23 | 17 | 12 | 12 | 15 | 13 | 17 | 18 | 29 |
| Age at first birth* | 27.0 (4.9) | 27.3 (4.7) | 26.7 (4.5) | 26.1 (4.1) | 26.1 (3.9) | 25.4 (3.3) | 26.2 (4.3) | 26.4 (4.5) | 27.0 (4.5) | 28.0 (5.2) |
| Parity* | 2.2 (0.9) | 2.2 (0.9) | 2.4 (1.0) | 2.4 (0.9) | 2.5 (0.9) | 2.4 (0.8) | 2.4 (1.0) | 2.5 (1.0) | 2.3 (0.8) | 2.0 (0.8) |
| History of benign breast disease (%) | 21 | 16 | 14 | 18 | 12 | 14 | 15 | 15 | 20 | 17 |
| Family history of breast cancer (%) | 10 | 9 | 7 | 7 | 9 | 8 | 8 | 7 | 8 | 12 |

Notes: Values are means (standard deviations) unless otherwise noted.

*Among parous women only
