# Supplementary material for: Adolescent and early adulthood inflammation-associated dietary patterns in relation to premenopausal mammographic density
Source: Breast Cancer Res. 2021 Jul 7;23:71. doi: 10.1186/s13058-021-01449-0 (PMC8261986; doi:10.1186/s13058-021-01449-0)
Supplement: Supplementary file 3 — Additional file 3: Table S3. Mean mammographic density phenotypes (95% confidence interval) by quintile of averaged dietary patterns (n=677) [file 13058_2021_1449_MOESM3_ESM.docx]

**Supplementary Table 3. Mean mammographic density phenotypes (95% confidence interval) by quintile of averaged dietary patterns (n=677)**

| **Pro-inflammatory dietary pattern** | | | | | | |
| --- | --- | --- | --- | --- | --- | --- |
|  | **Q1**  **(Lowest Inflammation)** | **Q2**  **(Low Inflammation)** | **Q3**  **(Moderate Inflammation)** | **Q4**  **(High Inflammation)** | **Q5**  **(Highest Inflammation)** | **P_trend_** |
|  | (n=154) | (n=161) | (n=130) | (n=122) | (n=110) |  |
| Percent mammographic density | | | | | | |
| Model 1^a^ | 43.0 (40.0-45.9) | 43.1 (40.1-46.0) | 41.3 (38.1-44.5) | 38.6 (35.8-41.4) | 37.3 (33.9-40.7) | 0.004 |
| Model 2^b^ | 41.2 (38.6-43.9) | 42.2 (39.8-44.6) | 41.2 (38.5-43.9) | 39.8 (37.4-42.3) | 39.7 (36.7-42.7) | 0.27 |
| Model 3^c^ | 41.1 (38.5-43.7) | 42.2 (39.8-44.6) | 41.1 (38.5-43.8) | 40.2 (37.9-42.5) | 39.6 (36.6-42.6) | 0.30 |
| Dense area in cm^2^ | | | | | | |
| Model 1^a^ | 44.2 (40.4-48.1) | 45.6 (42.2-49.1) | 41.9 (38.0-45.8) | 42.5 (39.0-45.9) | 44.8 (39.2-50.5) | 0.80 |
| Model 2^b^ | 44.1 (40.1-48.0) | 45.5 (42.1-49.0) | 41.9 (38.0-45.8) | 42.6 (39.1-46.0) | 45.1 (39.5-50.7) | 0.92 |
| Model 3^c^ | 43.8 (40.0-47.7) | 45.4 (42.0-48.9) | 42.0 (38.1-46.0) | 43.2 (39.8-46.6) | 44.8 (39.3-50.3) | 0.97 |
| Non-dense area in cm^2^ | | | | | | |
| Model 1^a^ | 65.2 (58.3-72.1) | 69.1 (62.6-75.7) | 68.8 (60.8-76.7) | 76.8 (68.7-84.9) | 84.6 (74.9-94.3) | 0.001 |
| Model 2^b^ | 71.2 (65.7-76.6) | 72.2 (67.5-76.9) | 69.2 (63.6-74.7) | 72.7 (66.9-78.5) | 76.7 (70.3-83.2) | 0.24 |
| Model 3^c^ | 71.5 (66.1-76.9) | 71.8 (67.1-76.5) | 69.0 (63.6-74.4) | 72.9 (67.2-78.7) | 76.8 (70.5-83.1) | 0.23 |
| **AHEI dietary pattern** | | | | | | |
|  | **Q1**  **(Least Healthy)** | **Q2**  **(Unhealthy)** | **Q3**  **(Moderately Healthy)** | **Q4**  **(Healthy)** | **Q5**  **(Most Healthy)** | **P_trend_** |
|  | (n=103) | (n=140) | (n=148) | (n=155) | (n=131) |  |
| Percent mammographic density | | | | | | |
| Model 1^a^ | 40.3 (37.0-43.6) | 39.6 (36.9-42.4) | 42.2 (39.4-45.1) | 41.7 (38.9-44.5) | 40.6 (37.4-43.8) | 0.64 |
| Model 2^b^ | 39.8 (37.0-42.6) | 40.8 (38.4-43.3) | 41.3 (38.8-43.7) | 41.1 (38.7-43.5) | 41.5 (38.8-44.1) | 0.43 |
| Model 3^c^ | 39.5 (36.7-42.4) | 40.9 (38.5-43.3) | 40.9 (38.5-43.4) | 41.5 (39.2-43.7) | 41.6 (39.1-44.1) | 0.28 |
| Dense area in cm^2^ | | | | | | |
| Model 1^a^ | 39.9 (35.8-44.0) | 45.3 (41.2-49.4) | 44.6 (41.1-48.1) | 45.2 (41.8-48.5) | 43.3 (38.8-47.8) | 0.42 |
| Model 2^b^ | 39.8 (35.7-44.0) | 45.4 (41.3-49.5) | 44.5 (40.9-48.0) | 45.1 (41.7-48.5) | 43.4 (38.9-47.9) | 0.41 |
| Model 3^c^ | 39.5 (35.3-43.7) | 45.2 (41.2-49.2) | 44.4 (40.9-47.9) | 45.3 (42.0-48.7) | 43.7 (39.4-48.0) | 0.26 |
| Non-dense area in cm^2^ | | | | | | |
| Model 1^a^ | 69.0 (59.7-78.3) | 74.3 (68.1-80.6) | 68.9 (62.3-75.6) | 71.6 (64.2-79.1) | 76.2 (67.7-84.6) | 0.39 |
| Model 2^b^ | 70.6 (64.8-76.5) | 70.3 (65.5-75.2) | 72.2 (67.3-77.0) | 73.6 (68.2-79.0) | 73.9 (68.1-79.6) | 0.30 |
| Model 3^c^ | 70.9 (65.0-76.7) | 70.1 (65.3-74.9) | 72.8 (67.9-77.8) | 72.9 (67.7-78.2) | 74.1 (68.3-80.0) | 0.32 |

^a^ Adjusted for adolescent and early adulthood total calorie intake and age at time of mammogram

^b^ Additionally adjusted for BMI at time of mammogram

^c^ Additionally adjusted for BMI at age 18 (kg/m^2^; cont.), adolescent physical activity (METs/week, quartiles), adolescent alcohol intake (drinker vs. non-drinkers), age at menarche (<12; 12; 13; 14+ years), age at first birth (AFB) and parity combined (Nulliparous; AFB <25 years, 1-2 kids; AFB 25+ year, 1-2 kids; AFB any age, 3+kids), biopsy confirmed benign breast disease (yes; no), first-degree family history of breast cancer (yes; no)

^d^ Trend test is based on the median of the category
